# Patent Ductus Arteriosus Persistence and Neurodevelopmental Outcomes: A Restrictive Treatment Approach Does Not Compromise Neurological Development

**DOI:** 10.1111/apa.70511

**Published:** 2026-03-20

**Authors:** Moses Kallenberger, Alina Bartling, Britta Hüning, Monia Vanessa Dewan, Johanna Bialas, Lisa Middendorf, Anne‐Kathrin Dathe, Ursula Felderhoff‐Müser, Anja Stein

**Affiliations:** ^1^ Department of Pediatrics I, Neonatology, Pediatric Intensive Care, Pediatric Infectiology and Pediatric Neurology University Hospital Essen, University Duisburg‐Essen Essen Germany; ^2^ Department of Pediatric Neurology, Centre for Neuromuscular Disorders, Centre for Translational Neuro‐ and Behavioral Sciences, University Hospital Essen, C‐TNBS University of Duisburg‐Essen Essen Germany; ^3^ Department of Health and Nursing, Occupational Therapy Ernst‐Abbe‐University of Applied Sciences Jena Jena Germany

**Keywords:** general movement assessment, magnetic resonance imaging, neurodevelopment, patent ductus arteriosus, transcerebellar diameter

## Abstract

**Aim:**

Optimal treatment strategies for patent ductus arteriosus (PDA) and their impact on neurodevelopmental outcomes remain controversial. This study investigates the influence of PDA duration on neurodevelopment under a restrictive treatment approach.

**Methods:**

Retrospective single‐center study of 171 preterm infants ≤ 32 weeks gestation with echocardiographically‐identified PDA (treated hemodynamically significant PDA—hsPDA, untreated PDA—ntPDA) or no PDA. Assessments included cerebral MRI at term‐equivalent age, General Movement Assessment at 8–16 weeks and Bayley Scales of Infant Development III at 22–26 months corrected age. Infants with severe IVH ≥ III° were excluded a priori to isolate PDA effects without major brain injury.

**Results:**

The hsPDA group represented infants with lower gestational age and birth weight, higher rates of bronchopulmonary dysplasia and longer hospitalization (all *p* < 0.001). Transcerebellar diameter differed significantly between groups (*p* = 0.004). PDA duration showed a statistical association with MRI‐detected IVH ≤ II° in the hsPDA subgroup (OR 1.01 per day, *p* = 0.05). Multivariate analysis did not confirm any significant impact of PDA group or duration on neurodevelopmental outcomes after adjusting for confounders.

**Conclusion:**

Gestational age and comorbidities, rather than longer PDA duration, determined neurodevelopmental outcomes in our cohort of very preterm infants without severe brain injury.

## Summary

- Optimal management of patent ductus arteriosus in preterm infants remains controversial, with limited evidence on neurodevelopmental outcomes following restrictive treatment.
- Patent ductus arteriosus duration was not independently associated with adverse neurodevelopmental outcomes at two years corrected age in very preterm infants without severe brain injury.
- These findings suggest that conservative patent ductus arteriosus management with surgical intervention only in selected cases does not compromise neurodevelopment at two years corrected age.

**Abbreviations**
Bayley‐III: Bayley Scales of Infant and Toddler Development III
BPD: bronchopulmonary dysplasia
CI: confidence interval
hsPDA: hemodynamically significant patent ductus arteriosus
IQR: interquartile range
IVH: intraventricular haemorrhage
MOS‐R: Motor Optimality Score‐Revised for General Movement Assessment
MRI: magnetic resonance imaging
noPDA: no patent ductus arteriosus
ntPDA: untreated patent ductus arteriosus
OR: odds ratio
PDA: patent ductus arteriosus
PVL: periventricular leukomalacia
SWI: susceptibility weighted imaging in MRI at term equivalent age
TAS: Total Abnormality Score in MRI at term equivalent age
TCD: transcerebellar diameter in MRI at term equivalent age
US: ultrasound

## 1 Introduction

Patent ductus arteriosus (PDA) represents one of the most common heart conditions in preterm infants, with incidence inversely related to gestational age [1]. PDA persistence is associated with significant morbidities including bronchopulmonary dysplasia (BPD), necrotizing enterocolitis and intraventricular or intracerebral haemorrhage [2]. Despite medical treatment availability, protective effects on mortality and morbidity remain unproven [3]. The optimal PDA management strategy remains controversial due to high spontaneous closure rates and conflicting evidence regarding treatment indications, timing and impact on neurodevelopment [4, 5, 6, 7, 8, 9]. Neurodevelopmental outcomes represent a particularly important but understudied aspect of PDA management. Recent studies suggest prolonged PDA duration may independently affect cognitive and motor outcomes and reduce cerebellar growth [10, 11].

This study investigated the relationship between duration of PDA and markers for neurodevelopmental outcomes in preterm infants ≤ 32 weeks gestation in a center with a restrictive PDA treatment approach.

## 2 Methods

### 2.1 Study Design and Population

306 preterm infants ≤ 32 weeks gestational age were admitted between January 2017 and September 2022. Of these, 237 infants underwent at least one echocardiography and were eligible for this retrospective single‐center study. Exclusion criteria included death before follow‐up (*n* = 36), loss to follow‐up (*n* = 10), chromosomal abnormalities and major congenital malformations (*n* = 7).

**FIGURE 1:**
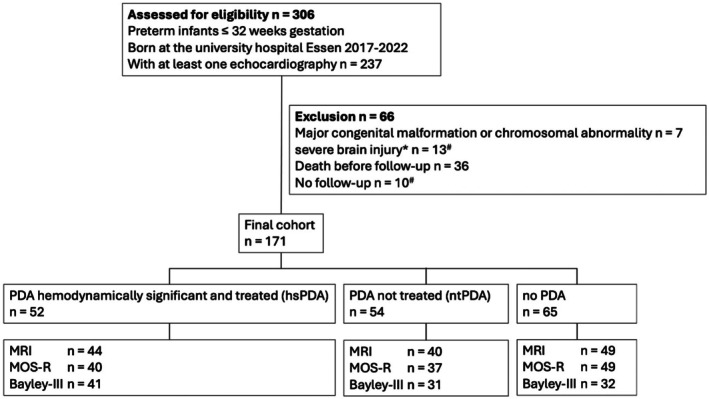
Patient flow. Bayley‐III, Bayley Scales of Infant Development III; MOS‐R, General Movement Motor Optimality Score‐Revised; MRI, Magnetic Resonance Imaging; PDA, Persistent Ductus Arteriosus, * intraventricular haemorrhage and/or intracerebral haemorrhage (IVH ≥ III°) and/or periventricular cysts. ^#^An additional 5 infants with severe brain injury were also lost to follow‐up and are counted in that category.

Severe brain injury represents an overwhelming independent predictor of neurodevelopmental outcomes that would obscure any potential duration‐related effects of PDA. A total of 18 infants with severe brain injury were identified: IVH III° without parenchymal haemorrhage (*n* = 1), additional intracerebral haemorrhage (*n* = 12) and/or periventricular cysts (*n* = 12). Some infants had multiple findings, accounting for the overlap. Three infants had periventricular cysts without any intraventricular or intracerebral haemorrhage. This group consisted of 9 male and 9 female infants with a median gestational age of 28.1 weeks (IQR 25.3–29.9) and a median birth weight of 0.97 kg (IQR 0.72–1.21). Regarding PDA status, 3 had no PDA, 4 were in the ntPDA group, and 11 were in the hsPDA group, of whom 2 required surgical ligation. Of these 18 infants, 13 were excluded due to brain injury and 5 due to loss to follow‐up. The final cohort comprised 171 infants (Figure 1). Severity of BPD in the cohort was defined according to Jensen et al. [12].

The study was approved by the Ethics Committee of the Medical Faculty of the University of Duisburg‐Essen (12–4981‐BO, 18–8388‐BO).

### 2.2 PDA Assessment and Treatment Protocol

Echocardiographic assessment was performed by a paediatric cardiologist within 72 h for infants ≤ 28 weeks and within 5 days for infants > 28 weeks gestational age. High risk criteria for hemodynamically significant PDA included left atrial and ventricular dilatation, reduced systolic function, non‐restrictive left‐to‐right shunting with peak velocity < 3 m/s and PDA diameter > 1.5 mm. Clinical criteria for treatment included high‐risk echocardiographic findings plus cardiac symptoms, respiratory symptoms, abdominal signs, or perfusion changes on ultrasound. Regular echocardiographic and careful clinical monitoring was applied if PDA remained open. Surgical ligation was only pursued if the PDA remained hemodynamically significant beyond 4 weeks of age, with persistent clinical symptoms and no further medical treatment options (either because of contraindications or failure of > 3 treatment courses). Infants were categorized as: treated hemodynamically significant PDA (hsPDA, *n* = 52, 30.4%), untreated PDA (ntPDA, *n* = 54, 31.6%) and no PDA (noPDA, *n* = 65, 38.0%). Definite PDA closure was defined as absence on echocardiography that was confirmed on all subsequent examinations (minimum 2 follow‐up echocardiograms at ≥ 1‐week intervals). If reopening occurred, the closure date was reset.

### 2.3 Neurodevelopmental Assessments

All infants ≤ 32 weeks gestation routinely undergo brain MRI at term‐equivalent age (3 Tesla, Skyra, Siemens). The Total Abnormality Score (TAS) originally developed by Kidokoro et al. [13] and modified by Dewan et al. [14] assesses brain injuries and development on a 0–32‐point scale. Additional quantitative measurements included biparietal width, interhemispheric distance and transcerebellar diameter (TCD). All measurements were performed by two investigators, blinded to PDA status.

General Movement Motor Optimality Score—Revised (MOS‐R) was assessed at 8–16 weeks corrected age by two advanced certified scorers [15]. Neurodevelopmental outcomes were assessed using Bayley Scales of Infant and Toddler Development III (Bayley‐III) at 22–26 months corrected age [16].

### 2.4 Statistical Analysis

The sample size was determined by the available cohort during the study period rather than a priori power calculation. Post hoc power calculations demonstrated 90% power to detect a 15‐point difference in Bayley‐III Motor Scores and 82% power to detect a 2 mm difference in TCD. Categorical variables are presented as frequencies and percentages. Continuous variables are presented as median with interquartile range (IQR) given non‐normal distributions. Non‐parametric tests were employed throughout the analysis given data distributions. Kruskal–Wallis H test and Chi‐square test were used to analyse differences between PDA groups. Exploratory pairwise comparisons used Mann–Whitney *U* test with Bonferroni adjustment. *p* ≤ 0.05 was considered nominally significant. Binary logistic regression was performed to identify factors associated with hsPDA. The dependent variable was hsPDA (vs. ntPDA/noPDA). Independent variables included gestational age, birth weight and BPD, selected based on clinical relevance. Model fit was assessed by Hosmer–Lemeshow test. Results are presented as odds ratios (OR) with 95% confidence intervals. Multivariable linear regression models were constructed with neurodevelopmental outcomes (Bayley‐III subscales, MOS‐R, TCD, TAS) as the dependent variable and PDA duration (in days) as the main predictor of interest. We reported only the analysis of the hsPDA subgroup, as PDA duration likely represents a clinically meaningful exposure only in the presence of hemodynamic significance. Models were adjusted for gestational age and birth weight as potential confounders. BPD was not included as a covariate, as it may represent a mediator on the causal pathway between prolonged PDA and neurodevelopmental impairment rather than a confounder. Model assumptions were verified through examination of residual plots and Q‐Q plots. Binary logistic regression also examined the association between PDA duration and IVH ≤ II° (MRI‐SWI) in the hsPDA subgroup, adjusted for gestational age and birth weight. Nagelkerke *R* ^2^ is reported. Although BPD showed a bivariate association with IVH, it was not independently associated with the outcome in the multivariable model and was therefore not retained in the final model. Model fit was assessed by Hosmer‐Lemeshow test. No interaction or non‐linear terms were included due to limited sample size.

Analyses were performed using IBM SPSS Statistics for iOS Version 31.0.1.0.

## 3 Results

### 3.1 Patient Characteristics

The cohort included 171 infants (47 multiples) with a median gestational age of 29.7 weeks (IQR 27.6–31.0), a median birth weight of 1.23 kg (IQR 0.90–1.52). Male infants comprised 48.5% of the cohort. Length of hospital stay ranged from 20 to 181 days (median 54 days, IQR 39–83) with a median gestational age at discharge of 38 weeks (IQR 36–39).

Infants with hsPDA had significantly lower median gestational age (27.2 weeks, IQR 25.4–28.4) compared to those with ntPDA (30.1 weeks, IQR 28.8–30.9; *p* < 0.001) and noPDA (30.9 weeks, IQR 29.5–31.4; *p* < 0.001). Birth weight followed the same pattern: hsPDA 0.86 kg (IQR 0.64–1.10), ntPDA 1.41 kg (IQR 1.04–1.61), noPDA 1.40 kg (IQR 1.14–1.66); with significant differences between hsPDA versus ntPDA and hsPDA versus noPDA *p* < 0.001. Gestational age and birth weight did not differ significantly between ntPDA and noPDA groups (*p* = 0.29 and *p* = 1.00). The hsPDA group had higher BPD rates (21.2%) compared to ntPDA (5.6%) and noPDA groups (1.5%; *p* < 0.001) and prolonged length of hospital stay (hsPDA 86 days (IQR 67–118), ntPDA 50 days (IQR 38–66), noPDA 42 days (IQR 31–60); *p* < 0.001) (Table S1). After adjustment for gestational age and birth weight, PDA group was not significantly associated with BPD rate (*p* = 0.45).

Binary logistic regression identified gestational age (OR = 0.65 per week, 95% CI 0.50–0.85, *p* < 0.001), birth weight (OR = 0.92 per 100 g, 95% CI 0.87–0.97, *p* = 0.002) and BPD (OR = 4.24, 95% CI 1.52–11.78, *p* = 0.006) as independently associated with hsPDA. The model demonstrated good discrimination (AUC = 0.86, 95% CI 0.81–0.91) and adequate calibration (Hosmer‐Lemeshow *χ* ^2^ = 7.14, df = 6, *p* = 0.31, Table S3). Additional demographic and clinical variables (Table S1) were tested but did not significantly improve model fit.

### 3.2 Neurodevelopmental Outcomes

Bayley‐III was performed at 24 months corrected age (IQR 23–24). There were no significant differences between PDA groups in cognitive (*p* = 0.82, *n* = 103) or language scales (*p* = 0.86, *n* = 76). Motor scales analysed across the three groups showed a trend towards significance (*p* = 0.06, *n* = 102); exploratory post hoc pairwise comparisons could not confirm significant differences. The largest observed difference was 7.2 points (hsPDA vs. noPDA, *p* = 0.06), below the clinically meaningful threshold of 15 points. Multivariable linear regression analysis revealed no correlation between PDA duration and these outcomes after adjusting for gestational age and birth weight in the hsPDA subgroup (cognitive scale *p* = 0.52, language scale *p* = 0.78, motor scale *p* = 0.65; Table S4).

MOS‐R showed no significant differences (*p* = 0.53, *n* = 126) with median values of 22.0 (IQR 20.0–24.0), 21.0 (IQR 20.0–23.5) and 22.0 (IQR 21.0–24.0) in the hsPDA, ntPDA and noPDA groups respectively (Table S2). No correlation with PDA duration was observed (*p* = 0.53) in multivariable linear regression in the hsPDA subgroup after adjustment for gestational age and birth weight (Table S4).

Data from MRI performed at a mean corrected age of 40.1 weeks (IQR 40.0–40.3) were available for 122–133 patients depending on the specific parameter analysed. Only TCD differed significantly between groups (*p* = 0.004, *n* = 129): hsPDA 52.2 mm (IQR 49.9–54.3), ntPDA 53.8 mm (IQR 51.7–55.7), noPDA 54.1 mm (IQR 52.2–55.9). Post hoc groupwise analysis revealed significantly smaller TCD in hsPDA versus noPDA patients (*p* = 0.004, 95% CI for difference 1.0–3.4 mm). TAS (*n* = 133) showed no associations with PDA status (*p* = 0.07, Table S2). In multivariable linear regression in the hsPDA subgroup, PDA duration was not independently associated with TCD (*p* = 0.17) or TAS (*p* = 0.87) after adjustment for gestational age and birth weight. For TCD, birth weight was the only significant predictor (*p* = 0.004); for TAS, gestational age was the only significant predictor (*p* = 0.02). Detailed regression results are presented in Table S4. While infants with severe brain injury (IVH ≥ III° and periventricular cysts) have been excluded from the study a numerical trend towards higher rates of IVH ≤ II° detected by MRI at term‐equivalent age in susceptibility weighted imaging (SWI) was observed in the hsPDA group (22.7%) compared to the ntPDA (12.5%) and noPDA group (8.2%). This did not reach statistical significance (*p* = 0.13). In the hsPDA group infants with IVH ≤ II° (10 of 44 infants with available MRI) had significantly longer median PDA duration compared to those without IVH (88 days [IQR 26.3–221.3] versus 7 days [IQR 5–65]; *p* = 0.01). This difference was not observed in the ntPDA group (*p* = 0.48). Within the hsPDA group, unadjusted binary logistic regression showed that longer PDA duration was significantly associated with increased frequency of IVH ≤ II° detected by MRI‐SWI (OR 1.01 per day, 95% CI 1.00–1.02, *p* = 0.02). After adjustment for gestational age and birth weight, this association remained with a stable effect (adjusted OR 1.01 per day, 95% CI 1.00–1.02, *p* = 0.05), while neither gestational age (*p* = 0.28) nor birth weight (*p* = 0.51) were independent predictors (Table S5). The adjusted model explained 41.9% of the variance in IVH occurrence (Nagelkerke *R* ^2^ = 0.42) and correctly classified 86.4% of cases. The Hosmer‐Lemeshow test indicated good model fit (*χ* ^2^(8) = 5.46, *p* = 0.71). This association could not be confirmed when investigating IVH detected by ultrasound (*p* = 0.19) or when analysing the ntPDA cohort separately (*p* = 0.93). There was no correlation of PDA duration in the hsPDA group with the severity of IVH on MRI SWI‐sequences (1/44 had hemosiderin deposits or post hemorrhagic cysts within caudo‐thalamic notch, 9/44 had hemosiderin deposits outside notch, along ventricle wall without ventricular dilatation).

### 3.3 Treatment Outcomes

Spontaneous ductal closure occurred in 94.4% (51/54) of the ntPDA patients, with a median closure time of 38 days of life (IQR 13–128 days, range 3–660 days). In three patients the PDA remained open at last follow‐up at an age of 22 months. Among hsPDA patients, 29 patients (55.8%) achieved closure following pharmacological treatment with a median closure time of 5 days (IQR 5–9 days, range 4–34), while 5.8% required surgical ligation (these 3 patients were operated on days 30, 42 and 71 of life respectively). In the remaining 20 patients (38.5%), spontaneous closure occurred during follow‐up at a median age of 146 days (IQR 86–217 days, range 58–456 days).

Pharmacological treatment included indomethacin in 43 cases (82.7%), ibuprofen in 11 cases (21.2%) and acetaminophen in 9 cases (17.3%). Ten patients received more than one pharmacological agent. The duration of medical treatment ranged from 2 to 29 days (median 2 days, IQR 3–6.8 days).

## 4 Discussion

This study investigated the relationship between PDA persistence and neurodevelopmental outcomes in preterm infants under restrictive treatment. Cerebellar development is particularly vulnerable in preterm infants and reduced volume at term‐equivalent age persists, making this parameter important for neurological prognosis [17]. Reduced TCD has been associated with pathological general movements [18] and a lower Mental Developmental Index in the Bayley Scales of Infant Development II and III [19] in very preterm infants. Group‐based analysis in our study revealed significant differences in TCD between PDA groups with a clear gradient: hsPDA < ntPDA < noPDA; but no differences in MOS‐R or any scales or subscales of Bayley‐III. This may reflect measurement timing at term‐equivalent age before catch‐up growth or suggest that modest reductions in cerebellar size do not necessarily translate to functional impairment at 2 years. Longer follow‐up may reveal delayed effects. When examining PDA duration as a continuous predictor through multiple regression analysis, no significant associations were found with any neurodevelopmental outcomes in either the complete cohort or the hsPDA subgroup. With adequate power (90%) to detect clinically meaningful 15‐point differences, the study found no PDA‐related effects exceeding this threshold. Our findings contrast with Lemmers et al. [10] and Kikuchi et al. [11], who reported significant associations between PDA duration and neurological outcomes and specifically reduced TCD, likely due to treatment philosophy and population differences.

While binary logistic regression analysis after adjustment for gestational age and birth weight showed a statistical association of PDA duration with mild IVH detected by MRI in the hsPDA subgroup (OR 1.01, 95% CI 1.00–1.02, *p* = 0.05), the clinical significance of this finding is likely limited. The confidence interval barely excludes unity and the effect size is minimal (1% increase in odds per day). Additionally, this association was only detectable for IVH diagnosed by MRI—not by cranial ultrasound as the standard clinical screening modality—suggesting it may reflect subclinical microhemorrhages without functional impact, as supported by the lack of correlation between IVH severity (IVH ≤ II°) and PDA duration, and the absence of differences in neurodevelopmental outcomes. The relationship with PDA duration was also not observed in the ntPDA cohort. Infants with severe IVH ≥ III°, intracerebral haemorrhage and PVL were excluded from the study due to their inherently worse neurological outcomes, multifactorial genesis and small numbers in our cohort. Among the remaining cases, MRI‐detected IVH severity did not correlate with PDA duration. Supporting this interpretation, IVH severity is incorporated into the TAS, which showed no association with either PDA group or PDA duration.

Our restrictive treatment approach, with high pharmacological treatment rates and low rates of surgical intervention (5.8%) in hemodynamically significant PDA, was not associated with compromised neurodevelopmental outcomes in infants without or with mild IVH. These results align with recent guidelines supporting conservative PDA management. The 2025 American Academy of Paediatrics clinical report emphasized that early routine treatment does not improve outcomes [1]. Our findings also align with the recent meta‐analysis by Buvaneswarran et al. [20], which demonstrated that active PDA treatment during the first two weeks increased death or severe BPD risk (RR 1.10, 95% CI 1.01–1.19) compared to expectant management.

Key limitations include temporal bias over the six‐year study period, selection bias from including only survivors, small sample size and a follow‐up period limited to 2 years. Our analysis revealed that infants with hemodynamically significant PDA requiring treatment were systematically more immature and had higher rates of comorbidities. This represents classic ‘confounding by indication’, where treatment decisions are influenced by factors that independently affect outcomes. Furthermore, the timing of PDA closure was not determined by structured regular echocardiography but rather from clinically indicated or routine exams at discharge or during follow‐up appointments. The exclusion of infants with severe IVH (≥ III°), intracerebral haemorrhage and periventricular leukomalacia introduces selection bias that may underestimate PDA‐associated morbidity. While necessary to isolate PDA's independent effects in this small cohort, this approach excludes the most severely affected infants in whom prolonged PDA may have contributed to brain injury through hemodynamic instability.

Our findings therefore apply specifically to preterm infants without severe brain injury and cannot be extrapolated to infants with IVH ≥ III°, intracerebral haemorrhage, or PVL. The observed association between PDA duration and mild IVH in the hsPDA subgroup might have been more pronounced had severe cases been included.

Future research should focus on standardized definitions for hemodynamically significant PDA, integration of biochemical biomarkers that may predict PDA persistence and treatment response [21], multi‐center prospective studies with standardized monitoring protocols and larger sample sizes with a full spectrum of brain injury severity, extended follow‐up through school age and investigation of specific subgroups most likely to benefit from intervention.

## 5 Conclusion

While an association between PDA duration and MRI‐detected mild IVH emerged in the hsPDA subgroup, PDA duration itself did not independently predict adverse neurodevelopmental outcomes when confounding factors were considered. The observed reduced TCD in the hsPDA group appeared mediated by gestational age and associated comorbidities, particularly bronchopulmonary dysplasia. In our cohort of preterm infants without severe brain injury, restrictive PDA management and hence longer duration of PDA was not associated with compromised neurodevelopmental outcomes until two years corrected age. However, the observational nature of this study precludes causal inference, and prospective randomized trials are needed to establish optimal treatment strategies.

The complexity of factors influencing neurological development in preterm infants underscores the need for continued research incorporating standardized assessment tools, biomarker integration and extended follow‐up periods.

## Author Contributions

**Moses Kallenberger:** conceptualization, data curation, formal analysis, writing – original draft, validation. **Alina Bartling:** data curation, validation, writing – review and editing. **Britta Hüning:** writing – review and editing, data curation, resources. **Monia Vanessa Dewan:** data curation, methodology, writing – review and editing. **Johanna Bialas:** data curation, writing – review and editing. **Lisa Middendorf:** data curation, writing – review and editing. **Anne‐Kathrin Dathe:** data curation, methodology, writing – review and editing. **Ursula Felderhoff‐Müser:** data curation, writing – review and editing. **Anja Stein:** conceptualization, data curation, formal analysis, methodology, writing – original draft, writing – review and editing, visualization, supervision, validation.

## Funding

The authors have nothing to report.

## Conflicts of Interest

The authors declare no conflicts of interest.

## Supporting information


**Table S1:** Comparison of patient characteristics between PDA groups.
**Table S2:** Comparisons of neurodevelopmental outcomes between PDA groups.
**Table S3:** Binary logistic regression model for associations with hsPDA.
**Table S4:** Multiple linear regression analyses for PDA duration and neurodevelopmental outcomes with adjustment for gestational age and birth weight in hsPDA subgroup.
**Table S5:** Binary logistic regression for PDA duration and IVH≤II° (MRI detected) with adjustment for gestational age and birth weight in hsPDA subgroup.

## Data Availability

The data that support the findings of this study are available from the corresponding author upon reasonable request.
