# Supplementary material for: Patent Ductus Arteriosus Persistence and Neurodevelopmental Outcomes: A Restrictive Treatment Approach Does Not Compromise Neurological Development
Source: Acta Paediatr. 2026 Mar 20;115(7):1461–6. doi: 10.1111/apa.70511 (PMC13250968; doi:10.1111/apa.70511)
Supplement: Supplementary file 1 — Table S1: Comparison of patient characteristics between PDA groups. Table S2: Comparisons of neurodevelopmental outcomes between PDA groups. Table S3: Binary logistic regression model for associations with hsPDA. Table S4: Multiple linear regression analyses for PDA duration and neurodevelopmental outcomes with adjustment for gestational age and birth weight in hsPDA subgroup. Table S5: Binary logistic regression for PDA duration and IVH≤II° (MRI detected) with adjustment for gestational age and birth weight in hsPDA subgroup. [file APA-115-1461-s001.docx]

**Table S1: Comparison of patient characteristics between PDA groups**

| Variable | All  (n=171) | hsPDA  (n=52) | ntPDA  (n=54) | noPDA  (n=65) | Group differences* | hsPDA vs noPDA^#^ | hsPDA vs ntPDA^#^ | ntPDA vs noPDA^#^ |
| --- | --- | --- | --- | --- | --- | --- | --- | --- |
| Gestational age  (weeks) | 29.7 [27.6-31.0] | 27.2 [25.4-28.4] | 30.1 [28.8-30.9] | 30.9 [29.5-31.4] | **< 0.001** | -3.7 [- 4.0- 2.7]  **< 0.001** | -2.9 [- 3.6- -2.2]  **< 0.001** | -0.8 [1.0-0.0]  0.29 |
| Birth weight  (kg) | 1.23 [0.90-1.52] | 0.86 [0.64-1.10] | 1.41 [1.04-1.61] | 1.40 [1.14-1.66] | **< 0.001** | -0.54 [-0.63- -0.38]  **< 0.001** | -0.55 [-0.61- -0.33]  **< 0.001** | 0.01 [-0.10-0.18]  1.0 |
| Invasive ventilation  (days) | 7 [1-15] | 19.5 [11.5-31] | 7 [1.8-11] | 2 [0-7] | **< 0.001** | 15.5 [12-21]  **< 0.001** | 13 [8-17]  **< 0.001** | 5 [1-5]  **0.03** |
| Length of stay  (days) | 54 [39-82] | 86 [67-118] | 50 [38-66] | 42 [31-60] | **< 0.001** | 44 [43-53]  **< 0.001** | 36 [26-46]  **< 0.001** | 7.5 [0-14]  0.30 |
| Male gender  (yes) | 83 (48.5%) | 29 (55.8%) | 33 (61.1%) | 21 (32.3%) | **0.003** | 1.7 [1.1-2.5]  **0.04** | 0.8 [0.4-1.7]  1.0 | 3.3 [1.5-7.1]  **0.006** |
| BPD  (yes) | 15 (8.8%) | 11 (21.2%) | 3 (5.6%) | 1 (1.5%) | **< 0.001** | 4.1 [1.5-11.7]  **0.02** | 4.6 [1.2-17.4]  0.08 | 3.3 [0.4-30.8]  0.77 |

Values displayed as median and interquartile range [IQR] for continuous variables or number and percent for categorial variables; significance for group differences* is by Kruskal-Wallis Test for continuous variables or by Chi-Squares test for categorial variables. Exploratory pairwise comparisons^#^ performed using Mann-Whitney U test for continuous variables (displayed as: difference between medians [95% Confidence Interval], Bonferroni adjusted significance p) or by binary logistic regression for categorial variables (displayed as Odds Ratio [95% Confidence Interval], Bonferroni adjusted significance p). Significance threshold p < 0.05 (bold).

Abbreviations: BPD – Bronchopulmonary dysplasia, PDA – Persistent Ductus Arteriosus, hsPDA – hemodynamically-significant PDA, ntPDA – non-significant untreated PDA, noPDA – no PDA

**Table S2. Comparisons of neurodevelopmental outcomes between PDA groups**

| Variable | All  (n=171) | hsPDA  (n=52) | ntPDA  (n=54) | noPDA  (n=65) | Group differences* | hsPDA vs noPDA^#^ | hsPDA vs ntPDA^#^ | ntPDA vs noPDA^#^ |
| --- | --- | --- | --- | --- | --- | --- | --- | --- |
| Ultrasound ≤ II° IVH (yes) | 25 (14.6%) | 11 (21.2%) | 8 (14.8%) | 6 (9.2%) | 0.19 | 1.6 [1.0-2.8]  0.23 | 1.5 [0.6-4.2]  1.0 | 1.7 [0.6-5.3]  1.0 |
| MRI TCD  (mm) | 53.2 [51.6-55-6]  (n=129) | 52.2 [49.9-54.3]  (n=43) | 53.8 [51.7-55.7]  (n=38) | 54.1 [52.2-55.9]  (n=48) | **0.004** | -1.9 [-3.3- -0.9]  **0.004** | -1.6 [-3.0- -0.1]  0.09 | -0.3 [-1.8-0.8]  1.00 |
| MRI IVH ≤ II°  (yes) | 19 (14.3%)  (n=133) | 10 (22.7%)  (n=44) | 5 (12.5%)  (n=40) | 4 (8.2%)  (n=49) | 0.13 | 1.8 [1.0-3.3]  0.18 | 2.1 [0.6-6.7]  0.68 | 1.6 [0.4-6.4]  1.0 |
| MRI TAS | 2 [2-4]  (n=133) | 2 [2-4.8]  (n=44) | 2.5 [2-5.5]  (n=40) | 2 [2-3]  (n=49) | 0.07 | 0 [0-1]  0.16 | -0.5 [0-0]  1.00 | 0.5 [0-1]  0.13 |
| MOS-R | 22.0 [20.8-24]  (n=126) | 22.0 [20.0-24.0]  (n= 40) | 21.0 [20.0-23.5]  (n= 37) | 22.0 [21.0-24.0]  (n= 49) | 0.53 | 0 [-1-1]  1.00 | 1 [0-2]  0.93 | -1 [-1-0]  1.00 |
| Bayley-III  Cognitive scale | 100 [85-110]  (n=103) | 100 [85-114]  (n=40) | 95 [85-115]  (n=31) | 105 [90-110]  (n=32) | 0.82 | -5 [-10-10]  1.00 | 5 [-10-10]  1.00 | -10 [-15-5]  1.00 |
| Bayley-III  Language scale | 94 [76-114]  (n=76) | 94 [75-114]  (n=35) | 91 [77-124]  (n=22) | 103 [75-114]  (n=19) | 0.86 | -9 [-15-11]  1.00 | 3 [-18-10]  1.00 | -12 [-15-18]  1.00 |
| Bayley-III  Motor scale | 92 [82-103]  (n=102) | 89 [76-100]  (n=41) | 96 [84-103]  (n=30) | 100 [89-106]  (n=31) | 0.06 | -11 [-17-0]  0.06 | -7 [-14-0]  0.42 | -4 [-10-4]  1.00 |

Values displayed as median and interquartile range [IQR] for continuous variables or number and percent for categorial variables; significance for group differences* is by Kruskal-Wallis Test for continuous variables or by Chi-Squares test for categorial variables. Exploratory pairwise comparisons^#^ performed using Mann-Whitney U test for continuous variables (displayed as: difference between medians [95% Confidence Interval], Bonferroni adjusted significance p) or by binary logistic regression for categorial variables (displayed as Odds Ratio [95% Confidence Interval], Bonferroni adjusted significance p). Significance threshold p < 0.05 (bold).

Abbreviations: MOS-R –Motor Optimality Score – Revised, IVH – intraventricular hemorrhage, MRI – Magnetic Resonance Imaging, Bayley-III – Bayley Scales of Infant Development III, PDA – Persistent Ductus Arteriosus, TAS – Total Abnormality Score, TCD – Transcerebellar diameter, hsPDA – hemodynamically-significant PDA, ntPDA – non-significant untreated PDA, noPDA – no PDA

## **Table S3. Binary logistic regression model for associations with hsPDA**

| Variable | OR | 95% CI | p |
| --- | --- | --- | --- |
| Gestational age (per week) | 0.65 | 0.50 – 0.85 | **< 0.001** |
| Birth weight (per 100g) | 0.92 | 0.87 – 0.97 | **0.002** |
| BPD | 4.24 | 1.52 – 11.78 | **0.006** |

Model statistics: AUC = 0.86 (95% CI: 0.81–0.91); Hosmer-Lemeshow χ² = 7.14, df = 6, p = 0.31. Significance threshold p < 0.05 (bold).

Abbreviations: BPD - bronchopulmonary dysplasia ,OR - odds ratio; 95% CI – 95% confidence interval; p – significance

## **Table S4: Multiple linear regression analyses for PDA duration and neurodevelopmental outcomes with adjustment for gestational age and birth weight in hsPDA subgroup**

| Variable | B | 95% CI | β | p | Adjusted R2 |
| --- | --- | --- | --- | --- | --- |
| MRI TCD | -0.01 | -0.02 – 0.003 | -0.20 | 0.17 | 0.29 |
| MRI TAS | -0.001 | -0.01 – 0.01 | -0.03 | 0.87 | 0.08 |
| MOS-R | 0.004 | -0.01 – 0.02 | 0.10 | 0.53 | 0.11 |
| Bayley-III Cognitive scale | 0.02 | -0.04 – 0.08 | 0.11 | 0.52 | 0.04 |
| Bayley-III Language scale | 0.01 | -0.06 – 0.08 | 0.05 | 0.78 | -0.08 |
| Bayley-III Motor scale | 0.01 | -0.04 – 0.07 | 0.08 | 0.65 | -0.03 |

All models adjusted for gestational age and birth weight. PDA duration (in days) was the main predictor of interest. Model fit for each outcome is indicated by the adjusted R². Significance threshold p < 0.05 (bold).

Abbreviations: MOS-R –Motor Optimality Score – Revised, MRI – Magnetic Resonance Imaging, Bayley-III – Bayley Scales of Infant Development III, TAS – Total Abnormality Score, TCD – Transcerebellar diameter, B – unstandardized regression coefficient; β – standardized regression coefficient; p – significance level; adj. R² – adjusted coefficient of determination (proportion of variance in the outcome explained by the model, adjusted for number of predictors)

**Table S5: Binary logistic regression for PDA duration and IVH ≤II° (MRI detected) with adjustment for gestational age and birth weight in hsPDA subgroup**

| Variable | OR (per day) | 95% CI | p |
| --- | --- | --- | --- |
| MRI IVH ≤II° | 1.01 | 1.00 - 1.02 | **0.05** |

Adjusted for gestational age and birth weight. Neither were independent predictors (p = 0.28 and p = 0.51 respectively), Model statistics: AUC = 0.84 (95% CI: 0.71–0.98); Nagelkerke R^2^ 0.42, Hosmer-Lemeshow χ² = 5.46, df = 8, p = 0.71

Abbreviations: MRI IVH ≤II° - intraventricular hemorrhage ≤ II° detected by Magnetic Resonance Imaging, OR - odds ratio; 95% CI – 95% confidence interval; p - significance
